# Real-Time Detection of Riboflavin Production by *Lactobacillus plantarum* Strains and Tracking of Their Gastrointestinal Survival and Functionality *in vitro* and *in vivo* Using mCherry Labeling

**DOI:** 10.3389/fmicb.2019.01748

**Published:** 2019-07-31

**Authors:** Mari Luz Mohedano, Sara Hernández-Recio, Alba Yépez, Teresa Requena, M. Carmen Martínez-Cuesta, Carmen Peláez, Pasquale Russo, Jean Guy LeBlanc, Giuseppe Spano, Rosa Aznar, Paloma López

**Affiliations:** ^1^Department of Microorganisms and Plant Biotechnology, Biological Research Center (CIB-CSIC), Madrid, Spain; ^2^Department of Microbiology and Ecology, University of Valencia, Valencia, Spain; ^3^Department of Biotechnology and Food Microbiology, Institute of Food Science Research (CIAL-CSIC), Madrid, Spain; ^4^Department of the Science of Agriculture, Food and Environment, University of Foggia, Foggia, Italy; ^5^Reference Centre for Lactobacilli (CERELA-CONICET), San Miguel de Tucumán, Argentina; ^6^Department of Preservation and Food Safety Technologies, Institute of Agrochemistry and Food Technology (IATA-CSIC), Paterna, Spain

**Keywords:** riboflavin, probiotic, lactic acid bacteria, *Lactobacillus plantarum*, fluorescent labeling

## Abstract

Some strains of lactic acid bacteria (LAB) produce riboflavin, a water-soluble vitamin of the B complex, essential for human beings. Here, we have evaluated riboflavin (B2 vitamin) production by five *Lactobacillus plantarum* strains isolated from *chicha*, a traditional maize-based fermented alcoholic beverage from north-western Argentina and their isogenic riboflavin-overproducing derivatives previously selected using roseoflavin. A direct fluorescence spectroscopic detection method to quantify riboflavin production in bacterial culture supernatants has been tested. Comparison of the efficiency for riboflavin fluorescence quantification with and without prior HPLC fractionation showed that the developed method is a rapid and easy test for selection of B2 vitamin-producing strains. In addition, it can be used for quantitative detection of the vitamin production in real time during bacterial growth. On the basis of this and previous analyses, the *L. plantarum* M5MA1-B2 riboflavin overproducer was selected for *in vitro* and *in vivo* studies after being fluorescently labeled by transfer of the pRCR12 plasmid, which encodes the mCherry protein. The labeling did not affect negatively the growth, the riboflavin production nor the adhesion of the strain to Caco-2 cells. Thus, *L. plantarum* M5MA1-B2[pRCR12] was evaluated for its survival under digestive tract stresses in the presence of microbiota in the dynamic multistage BFBL gut model and in a murine model. After exposure to both models, M5MA1-B2[pRCR12] could be recovered and detected by the pink color of the colonies. The results indicated a satisfactory resistance of the strain to gastric and intestinal stress conditions but a low colonization capability observed both *in vitro* and *in vivo*. Overall, *L. plantarum* M5MA1-B2 could be proposed as a probiotic strain for the development of functional foods.

## Introduction

Nowadays, vitamin requirement is usually adequately supplied by a balanced diet; however, the insufficient or reduced intake of some vitamins is an important cause of nutritional deficiencies which affects susceptible groups of the population (LeBlanc et al., 2011; Mensink et al., 2013). Vitamin B2 (riboflavin) is a water-soluble vitamin which is a central and crucial component of cellular metabolism, since it is the precursor of the coenzymes flavin mononucleotide (FMN) and flavin adenine dinucleotide (FAD) (LeBlanc et al., 2011). According to the European Food Information Council, the riboflavin “Recommended Daily Amount” (EU RDA) is 1.6 mg per day (EFSA NDA Panel, 2017) and this has to be ingested regularly, because human beings are unable to store this vitamin. Riboflavin production is a trait present in certain strains of lactic acid bacteria (LAB), which can be biotechnologically exploited to substitute the chemically synthesized vitamin in food fortification as a natural and economically viable strategy (Capozzi et al., 2012). Therefore, we screened LAB from traditionally fermented food to select strains with outstanding functional and technological traits (Carrizo et al., 2017; Jiménez et al., 2018). In addition, LAB strains which can synthesize B2 vitamin are ideal candidates to achieve vitamin overproduction by different strategies. In many Gram-positive bacteria, including some LAB strains, the synthesis of riboflavin is *via* the condensation of guanosine-5′-triphosphate and D-ribulose-5′-phosphate in seven enzymatic steps. This pathway is catalyzed by four proteins called RibA, RibG, RibB, and RibH, and their coding genes constitute the *ribGBAH* (*rib*) operon (reviewed by Thakur et al., 2015). It has been demonstrated that in *Bacillus subtilis* the expression of the *rib* operon is regulated by transcriptional attenuation at a “riboswitch” located at its 5′-untranslated region via FMN and riboflavin. This riboswitch contains the conserved RFN element, a region of the mRNA that can adopt two alternative secondary structures, one of which is the FMN-binding aptamer which also binds roseoflavin, a toxic riboflavin analog (Vitreschak et al., 2002; Winkler et al., 2002). It has been shown that *Lactococcus lactis* requires an intact *rib* operon for synthesis of riboflavin, and that transcription of the operon does not take place when riboflavin is present in the growth medium (Burgess et al., 2004). Moreover, these authors, as well as Sybesma et al. (2004) described the isolation of riboflavin-overproducing spontaneous mutants after exposure of *L. lactis* NZ9000 to roseoflavin, because by constitutive high intracellular production of riboflavin, the cells can overcome the challenge of this toxic compound (Kukanova et al., 1982). The overproducing phenotype was frequently associated with mutations in the conserved RFN regulatory region (Burgess et al., 2004). During the last decade resistance to roseoflavin has been a widely employed method to obtain B2 vitamin-overproducing LAB strains (Capozzi et al., 2011; Juarez del Valle et al., 2014). Importantly, these riboflavin-resistant strains are spontaneous, non-genetically modified organisms, and consequently should be acceptable for the production of vitamin B2-enriched foods (Burgess et al., 2006). In this context, five *Lactobacillus plantarum* strains isolated from Argentinian “chicha” (a fermented drink) (Elizaquível et al., 2015; Jiménez et al., 2018) were used to select B2-overproducing mutants carrying punctual mutations in the RFN region, upon their treatment with roseoflavin (Yépez et al., 2019). Moreover, determination of the mutants’ riboflavin production by HPLC analysis and evaluation of their technological properties indicated that they were good candidates for their use to produce cereal-based fermented food enriched with riboflavin and, of these, *L. plantarum* M5MA1-B2 showed the best performance (Yépez et al., 2019). In addition, the probiotic potential of this strain, by synthesizing riboflavin in the human digestive tract, deserves further investigation, which has been performed in this work. One desirable characteristic of the probiotic strains is their ability to tolerate the gastro-intestinal tract (GIT) stresses. It is usually tested *in vitro* using conditions representing different gastric residence times and acidic pHs and bile salt concentrations. An approach to increase representativeness for the *in vivo* gut environment without relevant ethical concerns is the dynamic simulation of the upper gastrointestinal along with the colonic microbiota such as in the BFBL simulator (Barroso et al., 2015). This simulator allows an accurate control of environmental parameters and can be used as a tool for studying the resistance of probiotics to the GIT stresses and their interaction with the human microbiota. However, these cannot reliably predict response of bacteria to a multifactorial *in vivo* system. For this purpose, prior to human interventions, animal models are needed and these should be designed to mimic the conditions of how the probiotics will be administered including the dosage and mode of administration (de Moreno de LeBlanc et al., 2016).

For testing the performance of potential probiotic bacteria *in vitro* and *in vivo* genetic tools could be used. Accordingly, plasmid vectors for fluorescent labeling of LAB and *Bifidobacterium* has been developed (Castro-Bravo et al., 2017) and reviewed (Landete et al., 2016). Among these tools, we have developed the pRCR12 plasmid (Russo et al., 2015), which allows LAB fluorescent-labeling with mCherry, and it has been validated for its use in *L. lactis*, *Lactobacillus acidophilus*, *Lactobacillus casei, Lactobacillus fermentum*, *L. plantarum*, *Lactobacillus sakei*, and *Pediococcus parvulus* (Mohedano et al., 2015; Russo et al., 2015; Nácher-Vázquez et al., 2017a; Perez-Ramos et al., 2017, 2018). Moreover, the fluorescence labeling of *L. fermentum, L. plantarum, L. sakei*, and *P. parvulus* with pRCR12 has allowed visualization of these bacteria in the zebrafish gut (Russo et al., 2015; Nácher-Vázquez et al., 2017b; Perez-Ramos et al., 2018). Therefore, pRCR12 plasmid has been used in this work to label the *L. plantarum* strains of interest prior to assaying *in vitro* and *in vivo* their performance under GIT conditions.

## Materials and Methods

### Bacterial Strains and Growth Conditions

The bacteria used in this work are presented in Table 1. The *L. plantarum* strains were routinely grown in de Man, Rogosa, Sharpe (MRS) broth (Pronadisa, Spain) at 37°C. The corresponding strains carrying the pRCR12 plasmid were grown in MRS broth containing chloramphenicol (Cm) at 10 μg/mL. In addition, to evaluate the riboflavin production, these strains were grown in a chemically defined medium (CDM) (Sánchez et al., 2008) lacking riboflavin (CDM-Rib), and to test the inducibility of expression of the *rib* operon the CDM medium was used supplemented with either riboflavin (CDM + Rib) or FMN (CDM + FMN) at 2 μg/mL. Solid media were prepared by addition of agar (Pronadisa) to the liquid broth at a final concentration of 1.5%.

**TABLE 1:** Bacteria used in this work.

**Bacteria**	**CECT no.**	**Plasmid**	**Antibiotic resistance**	**Characteristics**	**References**
*L. plantarum* M5MA1	8962			Bacterium isolated from Argentinian Chicha	Elizaquível et al., 2015
*L. plantarum* M9MM1	8963			Bacterium isolated from Argentinian Chicha	Elizaquível et al., 2015
*L. plantarum* M9MG6	8965			Bacterium isolated from Argentinian Chicha	Elizaquível et al., 2015
*L. plantarum* M9Y2	8966			Bacterium isolated from Argentinian Chicha	Elizaquível et al., 2015
*L. plantarum* M9MM4	8964			Bacterium isolated from Argentinian Chicha	Elizaquível et al., 2015
*L. plantarum* M5MA1-B2	9434			B2-overproducing strain selected by treatment with roseoflavine	Yépez et al., 2019
*L. plantarum* M9MM1-B2	–			B2-overproducing strain selected by treatment with roseoflavine	Yépez et al., 2019
*L. plantarum* M9MG6-B2	9435			B2-overproducing strain selected by treatment with roseoflavine	Yépez et al., 2019
*L. plantarum* M9Y2-B2	–			B2-overproducing strain selected by treatment with roseoflavine	Yépez et al., 2019
*L. plantarum* M9MM4-B2	9436			B2-overproducing strain selected by treatment with roseoflavine	Yépez et al., 2019
*L. plantarum* 90[pRCR12]	–	[pRCR12]	Cm^R^	Source of plasmid pRCR12	Russo et al., 2015
*L. plantarum* M5MA1[pRCR12]	–	[pRCR12]	Cm^R^	Strain fluorescently labeled with mCherry	This study
*L. plantarum* M5MA1-B2[pRCR12]	9402	[pRCR12]	Cm^R^	Strain fluorescently labeled with mCherry	This study

*CECT, Colección Españsola de Cultivos Tipo.*

### pRCR12 Plasmid Isolation and Transfer to *L. plantarum* Strains

Plasmid pRCR12 carries a transcriptional fusion of the pneumococcal P_x_ promoter and the *mrfp* gene, whose codons are optimized for expression in LAB, and which encodes a monomeric version of the red-fluorescent protein of *Discosoma* sp. (García-Cayuela et al., 2012). The pRCR12 plasmid was isolated from *L. plantarum* 90[pRCR12] using the “JetStar 2.0 Plasmid Purification kit” (Genomed, Löhne, Germany). This strain was grown in 25 mL of MRS supplemented with Cm at 10 μg/mL at 37°C, until stationary phase (1 × 10^9^ colony forming units (cfu)/mL). The bacteria were sedimented by centrifugation at 9,300 × *g* for 10 min at 4°C and washed with 10 mL of phosphate buffer saline (PBS, pH 7.4). Then, plasmid isolation was performed as described in the kit protocol, eluting the plasmid DNA in 160 μl at approximately 100 μg/mL. For transfer of pRCR12 to *L. plantarum* strains, 0.5 μg of the plasmid were used for electroporation (25 μF, 1.3 kV, and 200 Ω in 0.1 cm cuvettes) as previously described (Berthier et al., 1996) and transformants were selected in MRS-agar plates supplemented with Cm at 10 μg/mL.

### Analysis of the RFN Element

Chromosomal DNA from *L. plantarum* wild-type strains and their derivatives were obtained with the Microbial DNA extraction kit (Cabru, Milan, Italy) according to the manufacturer’s instructions. Quantity and quality of genomic DNA were assessed using a BioTek Eon spectrophotometer (BioTek, Winooski, VT, United States) and by visualization on 0.8% agarose gel. The primers RFNFpl (CAGCGCCTTGTTTGAT) and RFNR (TGGCCGTCTTTGACTA) (Macrogen, Madrid, Spain) were used to amplify a 649-bp fragment including the *rib* regulatory region. The PCR were carried out in a 25-μL volume reaction containing 20 ng of genomic DNA, 5 μL of 5× HotStar HiFidelity PCR Buffer, 0.2 nm of each primer, and 2.5 U/μL of the HotStar HiFidelity DNA Polymerase (Qiagen, Hilden, Germany). The thermal profile of the PCR was as follows: 95°C for 5 min, 35 cycles of 95°C for 30 s, 53°C for 45 s, 72°C for 75 s, and a final extension at 72°C for 7 min. Clean-up was performed by using a QIAquick PCR purification kit (Qiagen), and quantification and purity of the amplicons were determined spectrophotometrically and by visualization on 1.2% agarose gel. Sequencing was performed according to the EZ-seq service (Macrogen). Multiple sequence alignments of the RFN were performed using the Clustal Omega program^1^.

### Detection and Quantification of Bacterial Culture Fluorescences

#### Simultaneous Measurement of Cell Growth and Fluorescence

Sterile 96-Well Optical White w/Lid Cell Culture (Thermo Fisher Scientific, Rochester, NY, United States) were used to monitor simultaneously and in real time the growth and the fluorescence of *L. plantarum* cultures with a Varioskan Flask System (Thermo Fisher Scientific, Waltham, MA, United States).

Growth and the mCherry red fluorescence were spectrofotometrically determined as follows: overnight cultures grown in MRS were diluted in fresh medium to give an optical density at a wavelength of 600 nm (OD_600_) of 0.1 and 200 μL of each culture were analyzed in triplicate during growth in real time in the microtiter plate. The OD_600_ of the cultures was measured as well as the emission of the mCherry fluorescence at a wavelength of 610 nm upon excitation at a wavelength of 587 nm.

Growth and riboflavin fluorescence were spectro- fotometrically determined as follows: overnight cultures grown in MRS medium were sedimented by centrifugation at 9,300 × *g*, 10 min at room temperature, and resuspended in either CDM-Rif, CDM + Rib or CDM + FMN media to an initial OD at a wavelength of 480 nm (OD_480_) of 0.1 and three 200 μL aliquots of each culture were analyzed as indicated above. The cell growth was monitored by measuring the OD_480_ and the riboflavin fluorescence upon excitation at a wavelength of 440 nm and detection of emission at a wavelength of 520 nm.

In both cases, the experiments were performed in triplicate by incubating in the Varioskan equipment at 37°C and measuring OD and fluorescence at 30 min intervals.

#### Quantification of Riboflavin Levels by Fluorescence in Culture Supernatants

To carry out the riboflavin quantification, a calibration curve was made to correlate the fluorescence emitted by riboflavin solutions at 520 nm with the concentration of the compound dissolved therein (Supplementary Figure S1). To this end, serial dilutions of a solution of riboflavin in CDM at 10 mg/mL were performed, and aliquots of 0.2 mL of each dilution were analyzed in a 96-well plate polypropylene non-sterile w/o lid Black (Nalge Nunc, Rochester, NY, United States), with an excitation wavelength of 440 nm and an emission wavelength of 520 nm.

Quantification of riboflavin produced by the LAB strains was performed by measuring the fluorescence in the supernatants of cultures grown in CDM medium. To this end, CDM medium was inoculated at OD_480_ of 0.1 with cells recovered from MRS overnight cultures and resuspended in fresh medium. After 24 h incubation at 37°C, OD_480_ was measured., bacteria were sedimented by centrifugation and 0.2 mL aliquots of culture supernatants were used to measure fluorescence as described above. These determinations were made in duplicate. Riboflavin concentration was estimated by interpolation of fluorescence values in the calibration curve.

### Determination of Riboflavin Concentration After HPLC Fractionation

*Lactobacillus plantarum* strains were grown overnight in MRS at 37°C. Then, cells were sedimented by centrifugation at 9,300 × *g*, 10 min, at room temperature and resuspended in CDM to achieve an OD_480_ of 0.1, which corresponded to 2.1 × 10^7^ ± 0.4 cfu/mL. After incubation at 37°C for 24 h, bacteria were removed by centrifugation as above and the supernatants were recovered and subjected to HPLC fractionation and fluorescence identification and quantification of riboflavin according to the previously described procedure (Jakobsen, 2008). Briefly, chromatographic analyses were performed by using a HPLC equipped with a degasser system with nitrogen, a binary pump, and a fluorescence detector (Agilent-1100 Series, Palo Alto, CA, United States), and a Zorbax Eclipse Plus C 18 [4.6 × 150 mm, 5 μm internal diameter (i.d.)] analytical column with a pre-column Zorbax ODS (4.6 × 12.5 mm, 5 μm i.d.) (Agilent Technologies). Signals were recorded by a ChemStation computer software (Agilent, Palo Alto, CA, United States). HPLC analyses were carried out by an isocratic elution at 1 mL/min, using as mobile phase a methanol/water (35:65 v/v) mixture. The eluate was monitored by a fluorescence detector set at an excitation wavelength of 440 nm and an emission wavelength of 520 nm. Spectral analyses of standard were performed in order to determine LOD and LOQ. The limits of detection and quantification of riboflavin were 0.03 and 0.15 mg/L, respectively.

### Fluorescence Microscopy

Exponential cultures of the *L. plantarum* strains were sedimented and resuspended in PBS pH 7.2 to obtain a fivefold concentrated suspension. Then, without fixing, the suspensions (10 μL) were used for phase contrast and fluorescent microscopy analysis with a Leica DM1000 model microscope (Leica Microsystems, Mannheim, Germany) with a light source EL6000 and the filter system TX2 ET for detection of mCherry fluorescence, respectively. The microscope was connected to a DFC3000G camera (Leica Microsystems) with a CCD sensor. The image analysis was performed using Leica Application Suite X Software (Leica Microsystems).

### Adhesion of *L. plantarum* Strains to Caco-2 Cells

The Caco-2 human enterocyte cell line, obtained from the cell bank at CIB, was seeded in 24-well tissue culture plates (Falcon Microtest^TM^, Becton Dickinson, Franklin Lakes, NJ, United States) at a final concentration of 1 × 10^5^ cell/ mL and grown as mono-layers of differentiated cells for 14 days as previously described (Nácher-Vázquez et al., 2017a). Cell concentrations were determined as previously described (Garai-Ibabe et al., 2010).

To test the adhesion, overnight cultures of the *L. plantarum* strains grown in MRS were diluted to give an OD_600_ of 0.1 at 37°C and incubated until the end of the exponential phase. Then, bacteria were sedimented as indicated above, resuspended in Dulbecco’s Modified Eagle Medium (DMEM, Invitrogen) and added to Caco-2 cells at a concentration of 5 × 10^9^ cfu/mL. After incubation for 4 h at 37°C in an atmosphere containing 5% CO_2_, unattached bacteria were removed by three washings with 0.5 mL of PBS pH 7.2. Then, Caco-2 cells were detached from the well by incubating with 0.1 mL of 0.05% (w/v) trypsin-EDTA for 7 min at 37°C. The reaction was stopped by addition of 0.5 mL of PBS pH 7.2. The number of cell-associated bacteria was determined in two ways: (i) by plating for all strains and (ii) by measuring the mCherry fluorescence in the case of strains carrying the pRCR12 plasmid. In the second case, calibration curves correlating cfu/mL (determined by plating) and fluorescence (Supplementary Figure S2) were used to establish the adhesion levels. Three independent adhesion assays were performed for each strain and each sample was tested in duplicate.

### Dynamic Simulation of the GIT Conditions and Feeding With *L. plantarum* M5MA1-B2[pRCR12]

The BFBL gut model is a four-stage reactors system intended to simulate *in vitro* the small intestine (SI) and the microbial conditions of three regions (R1, R2, R3) of the human colon. At the beginning of the experiment, the colon reactors were all simultaneously inoculated with the same fecal sample from a healthy human volunteer. The colonic microbiota was allowed to stabilize, reaching the steady state after 2 weeks (stabilization period). The inoculum preparation and the nutritive medium composition were essentially as described by Barroso et al. (2015). After stabilization of the colonic microbiota, *L. plantarum* M5MA1-B2[pRCR12] was daily administered to the BFBL model (test period) during five consecutive days (in average 9.8 log cfu/day). Late exponential bacterial cultures were sedimented as above and resuspended in nutrient medium adjusted to pH 2 and added to the SI vessel. Simultaneously, the SI vessel received additional nutrient medium (pH 2) and the pancreatic juice containing a mix of bile salts, NaHCO_3_ and pancreatin from porcine pancreas, as described previously (Barroso et al., 2015), that neutralized the acidic pH. After 2 h of incubation at 37°C, the whole content of the vessel was automatically transferred to the first colon compartment (R1) at a flow rate of 5 mL/min. The transit of colonic content between the R1, R2, and R3 compartments was controlled with level sensors that maintain their volumes at 250, 400, and 300 mL, respectively. The whole experiment was repeated twice using fecal samples from two different volunteers.

All the vessels were maintained under anaerobic conditions by flushing N_2_ during the feeding and incubation in the SI vessel and during the transit of the gut content between the colon reactors. Samples were collected at regular time points (7, 9, 11, and 14 days of the stabilization period and every day during the test period) from the vessels and stored at −20°C until further analysis. Also, a 3-days washout sample (a period without the addition of *L. plantarum* M5MA1-B2[pRCR12]) was analyzed. Microbiological plate count analyses were performed at the time of sampling.

#### Microbiological Analyses

Viable *L. plantarum* M5MA1-B2[pRCR12]) counts were determined by plating onto agar MRS at 37°C for 48 h. Total bacteria and *Lactobacillus* numbers were quantified by qPCR using SYBR green methodology in a ViiA7 System (Life Technologies) using primers 968 F and UNI 1401 R (Nübel et al., 1996) and LabF362 and Lab-667-R (Rinttilä et al., 2004), respectively. Primers RibG-F (5′-AGGTCGGTGCGGTATTAGTCAAAG-3′) and RibG-R (5′-ACGCGCCTGTTCTGGTGTG-3′) were designed from the *L. plantarum* M5MA1-B2 genome sequence (Genbank accession number CAADEV010000001-CAADEV010000118) using the PrimerSelect tool of DNASTAR (Lasergen 14), and were used to quantify copies of the *ribG* gen in the *L. plantarum rib* operon for riboflavin biosynthesis (Capozzi et al., 2011).

### Analysis of SCFA and Ammonium

Samples from the R1, R2, and R3 compartments were centrifuged (10,000 × *g*, 10 min) and the supernatants analyzed by HPLC as described earlier (Barroso et al., 2015). Briefly, samples (20 μL) were injected on a HPLC system (Jasco, Tokyo, Japan) equipped with a UV-975 detector. SCFA were separated using a Rezex ROA Organic Acids column (Phenomenex, Macclesfield, United Kingdom) using 5 mM sulphuric acid as mobile phase. The elution profile was monitored at 210 nm and the identification of the peaks was carried out by comparing the retention times of target peaks with those of the standards: acetic, propionic, butyric, formic, succinic, and lactic acids. Calibration curves of these acids were carried out in the concentration range from 1 to 100 mM. Ammonium was determined using the Nessler’s reagent (Sigma) as previously described (Doo et al., 2017).

### *L. plantarum* M5MA1-B2[pRCR12] Survival in an *in vivo* Murine Model

*Lactobacillus plantarum* M5MA1-B2[pRCR12] was activated twice in MRS containing Cm at 10 μg/mL. After the second activation, the strain was washed twice with sterile saline solution (0.85% w/v NaCl) to remove any traces of the antibiotic and resuspended to its original volume in the same solution. Conventional adult BALB/c mice (male, 5 weeks old, weighing 25 ± 3 g) were obtained from the inbred animal facilities at the Centro de Referencia para Lactobacilos (CERELA-CONICET, San Miguel de Tucumán, Argentina). The animal protocol was pre-approved by the Animal Protection Committee of CERELA (protocol no. CRL-BIOT-LT-20142/A), and all experiments complied with the current laws of Argentina for the use of experimental animals. Each animal (21 in total) received a single intragastric (IG) gavage of 100 μl of the bacterial suspension (corresponding to 1 × 10^6^ cfu/animal) or the same amount of saline solution for the control group (consisting of seven animals). After administration, three animals that received bacterial suspensions and one animal from the control group were sacrificed at 0.5, 2, 4, 8, 24, and 36 h post-administration. Small and large intestines were aseptically removed, 2 mL sterile saline solution was added and tissues and their contents were homogenized. Serial dilutions in saline solution of the homogenates were plated on MRS-agar containing Cm and incubated at 37°C during 48 h and pink colonies were counted. Animal live weights were determined every day.

### Statistical Analysis

In the Caco-2 cells adhesion assays, and in the experiments with the murine model, the data are expressed as a mean ± standard deviation calculated from three independent experiments. The data were subjected to one-way analysis of variance (ANOVA) by using the SAS software. Tukey’s test was applied to determine the significant differences between the variables at *p* ≤ 0.05. Student’s *t*-test (*p* ≤ 0.05) was applied for comparison of *L. plantarum* M5MA1-B2[pRCR12] viable counts and qPCR, SCFA and ammonium results between stabilization (control) and test periods in the colon reactors.

## Results and Discussion

### Comparative Detection and Quantification of Riboflavin by Direct Fluorescence Measurement With and Without Previous HPLC Fractionation

Riboflavin can be detected quantitatively by measurement of its fluorescent emission at 520 nm after excitation at 440 nm. Commonly, samples containing vitamin B2 are subjected to HPLC fractionation prior to its detection. Therefore, the riboflavin production by five *L. plantarum* strains from Andean origin and their isogenic riboflavin-overproducing strains, selected by treatment with roseoflavin, was investigated by this method. Bacteria were grown in defined CDM-Rib medium lacking riboflavin and fluorescent components that could interfere with the vitamin B2 quantification. Analysis of the culture supernatants grown in CDM-Rib (lacking B2 vitamin) revealed, as previously observed (Yépez et al., 2019), that riboflavin-overproducing strains produced and secreted B2 vitamin at high level in a range from 1.3 to 3.2 mg/L and ith a specific concentration referred to the biomass (estimated from the OD_480_) varying from 0.71 to 0.98, this calculation showing that the M9MG6-B2 strain has the highest specific production (Table 2). In addition, vitamin B2 production by the wild-type strains was not detected by the HPLC analysis. Therefore, detection of riboflavin in supernatants of cultures grown in CDM-Rib was attempted by direct fluorescence measurement. For quantification, a standard curve was generated using vitamin B2 solutions dissolved in CDM-Rib (Supplementary Figure S1). The results depicted in Table 2 revealed that, in the culture supernatants of the riboflavin-overproducing derivative strains, the levels of the vitamin B2 were almost identical (for most of them) to those observed by the HPLC-fluorescence detection method, showing that in the presence of high levels of riboflavin direct quantification of this compound in culture supernatants could be performed. In addition, the more sensitive detection method by direct fluorescence measurement permitted the quantification of the levels of vitamin B2 secreted by the parental wild-type strains (Table 2). In previous studies of *L. plantarum* riboflavin-overproducing strains selected by exposure to roseoflavin, it was shown that they contain mutation at the RFN region (Burgess et al., 2006), Therefore, the DNA sequence of this region in the *L. plantarum* strains studied in this work was determined. All the wild-type strains showed the same sequence and punctual mutations were observed in the RFN region of the five riboflavin-overproducing strains (Supplementary Figure S3 and Table 2). Therefore, the effect of the mutations harbored by the B2-overproducing strains at the RFN element on the riboflavin secretion levels was estimated. To this end, ratios between concentrations of the specific riboflavin secreted by the overproducing and by its corresponding wild strain were calculated (Table 2). The highest 18-fold increase was observed for M9Y2-B2, carrying the mutation G114A, versus M9Y2. Furthermore, a similar fold increase of 7.5 and 6.5 was detected for M5MA1-B2 and M9MM1-B2 strains, respectively, which carry the same mutation G19A, even when their specific riboflavin production varied from 0.9 to 0.52.

**TABLE 2:** Analysis of riboflavin production of the indicated *L. plantarum* strains.

	**Determination after HPLC**			**Direct determination**		
**Bacteria**	**Concentracion of riboflavin (mg/L)**	**Specific concentration of riboflavin ([Riboflavin]/A280 nm)**	**Concentracion of riboflavin (mg/L)**	**Specific concentration of riboflavin ([Riboflavin]/OD_280_)**	**Mutation effect (fold increase)**	**Mutation in the RFN region**
M5MA1-B2	3.10 ± 0.31	0.90	3.33 ± 0.58	0.90	7.5	G19A
M9MM1-B2	2.44 ± 0.20	0.76	1.59 ± 0.08	0.52	6.5	G19A
M9MG6-B2	3.20 ± 0.40	0.98	3.35 ± 0.54	0.98	10.9	G19C
M9Y2-B2	1.30 ± 0.16	0.71	1.16 ± 0.13	0.72	18.0	G114A
M9MM4-B2	3.22 ± 0.20	0.96	3.04 ± 0.19	0.95	11.9	G125A
M5MA1	ND	–	0.32 ± 0.29	0.12		
M9MM1	ND	–	0.17 ± 0.11	0.08		
M9MG6	ND	–	0.22 ± 0.18	0.09		
M9Y2	ND	–	0.08 ± 0.05	0.04		
M9MM4	ND	–	0.19 ± 0.03	0.08		

*Riboflavin present in cultures supernatants was determined by measurement of its fluorescence directly or after HPLC fractionation. The OD_480_ of all the cultures was 2.7 ± 0.7, besides those for M9Y2 and M9Y2-B2 that was 1.5 ± 0.5. ND, riboflavin concentration below the detection (0.03 mg/L) or quantification (0.15 mg/L) limits. The mutation effect was calculated as the ratio of the specific concentration of vitamin B2 secreted by the riboflavin-overproducing and its isogenic riboflavin–producing strain.*

Finally, it should be stated that, in principle, the level of riboflavin secreted by the wild-type strains should be measurable by the HPLC method, since some of them were slightly higher than its lower quantification limit. Therefore, the lack of detection by this method could be due to some loss of the vitamin present in the culture supernatants during the purification steps or to the fact that without HPLC fractionation the three flavins (riboflavin and their two metabolic products FMN and FAD) could be detected together. The two hypotheses are feasible, but nevertheless our results indicate that the measurement of fluorescence could be used to study flavin-producing strains in real time.

### Comparative Analysis of Riboflavin Production by *L. plantarum* M5MA1-B2 and M5MA1 Strains

**FIGURE 1:**
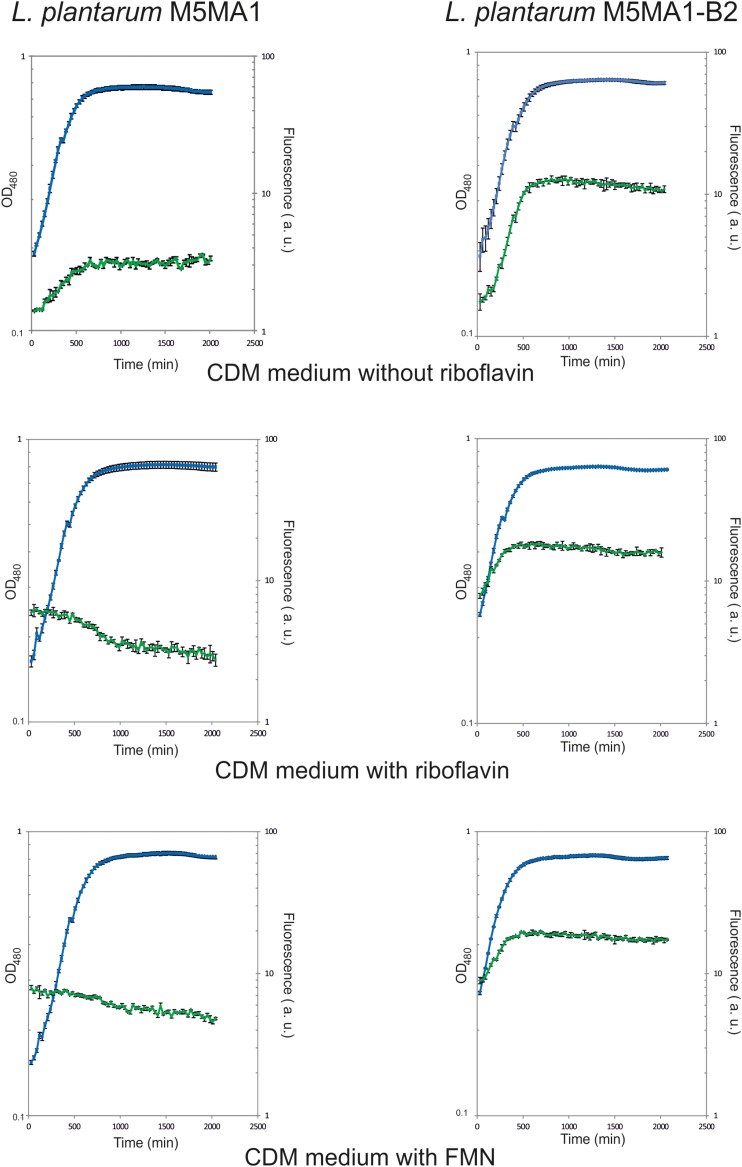
Detection during growth of riboflavin production by *L. plantarum* M5MA1 and M5MA1-B2. Bacteria were grown in CDM medium without riboflavin, or supplemented with either riboflavin or FMN both at a concentration of 2 μg/mL. The growth of cultures (blue) was monitored by measurement of OD_480_. Fluorescence emission of riboflavin (green) was recorded at 520 nm after excitation at a wavelength of 440 nm.

On the basis of its high production of riboflavin (>3 μg/mL) and its previously detected technological properties (Jiménez et al., 2018; Yépez et al., 2019), *L. plantarum* M5MA1-B2, together with its isogenic parental M5MA1 strain, were selected to further investigate riboflavin production. Fluorescence emission as well as OD_480_ of the cultures were measured in real time during growth (Figure 1). The CDM media containing either riboflavin or FMN or lacking both flavins were tested, since the presence of these compounds seem to be involved in the regulation of the *rib* operon expression in other Gram-positive bacteria including *L. lactis* (Winkler et al., 2002; Burgess et al., 2004). The media were supplemented with either riboflavin or FMN at 2 μg/mL, concentration at which production or consumption of the flavins during growth could be detected. Both strains grew similarly, in the three media tested, no decrease in the generation time (Gt) of the M5MA1-B2 versus M5MA1 (3.94 ± 0.67 h versus 3.7 ± 0.55) was observed in CDM-Rib medium. In addition, the cultures of the riboflavin-overproducing strain showed an increase of fluorescence independently of the medium tested, indicating a constitutive synthesis of the vitamin B2. By contrast, the cultures of the parental M5MA1 strain grown in either CDM + Rib or CDM + FMN medium showed a decrease of the initial fluorescence, indicating a consumption of the flavins. Moreover, as expected for an inducible expression of the *rib* operon, in cultures of the M5MA1 strain grown in CDM-Rib medium, lacking flavins, an increase of fluorescence was observed. Furthermore, as expected, the final levels of fluorescence of the M5MA1 cultures were approximately threefold lower than that of the equivalent M5MA1-B2 cultures. Therefore, these results confirmed that direct measurement of fluorescence of cultures grown in CDM defined medium can be used to detect and to investigate the performance of riboflavin-producing strains.

### Construction and Testing of the Recombinant Fluorescently Labeled *L. plantarum* M5MA1[pRCR12] and M5MA1-B2[pRCR12] Strains

**FIGURE 2:**
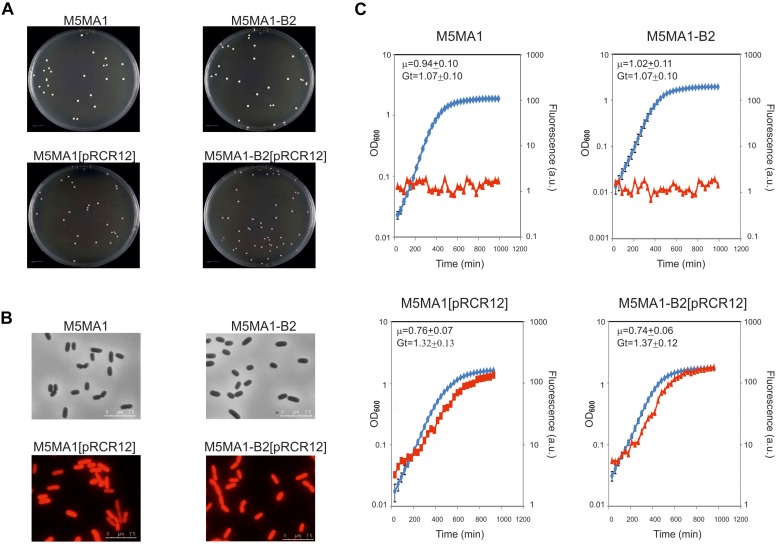
Comparative analysis of the *L. plantarum* M5MA1 and M5MA1-B2 carrying or lacking pRCR12. **(A)** Colony phenotypes of the parental and recombinant strains. **(B)** Overlays of phase contrast and fluorescence images of the four *L. plantarum* strains. Visualization of bacteria by optical microscopy with an objective of 100×. **(C)** Analysis of bacterial growth (blue) monitored by measurement of OD_480_ and their mCherry fluorescence emission (red) 610 nm upon excitation at a wavelength of 587 nm.

Strain M5MA1-B2 had previously proved efficiency for *in situ* riboflavin-fortification applications in functional foods (Yépez et al., 2019). However, its potential as a probiotic strain has not been previously investigated. Therefore, with the aim of analyzing its performance under digestive tract conditions the riboflavin-overproducing strain as well as its isogenic riboflavin-producing M5MA1 strain were fluorescently labeled by transfer of the pRCR12 plasmid, which encodes the mCherry protein. The choice of this vector was based on: (i) its efficiency to confer fluorescence to the *L. plantarum* strains (Russo et al., 2015), (ii) the wavelength for maximum fluorescence emission of the mCherry (610 nm), which does not overlap that of the flavins (520 nm), and (iii) this labeling permits bacterial detection even during growth in rich medium, such as MRS (Russo et al., 2015). Thus, as expected, cultures of the recombinant M5MA1-B2[pRCR12] and M5MA1[pRCR12] strains grown in MRS but not those of their parental strains: (i) generated pink colonies (Figure 2A), (ii) contained red fluorescent bacteria detected by fluorescent microscopy (Figure 2B), and (iii) emitted red fluorescence during growth detected by fluorescent spectroscopy (Figure 2C). In addition, the use of the recombinant strains carrying the pRCR12 plasmid instead of the corresponding non-labeled strains requires that the genetic manipulation did not drastically affect the bacterial growth. Thus, the growth of the four *L. plantarum* strains in liquid medium was analyzed and the growth rate at the exponential phase was calculated (Figure 2C). The results revealed that growth performance of the cultures in MRS medium was not significantly affected by the fluorescent labeling, since values of Gt of 1.32 h or 1.37 h versus 1.07 h or 0.99 h for isogenic strains carrying or not carrying pRCR12 were detected. Furthermore, concerning to flavin production and consumption in CDM medium, M5MA1-B2[pRCR12] and M5MA1[pRCR12] behaved like M5MA1-B2 and M5MA1, respectively (Supplementary Figure S4 versus Figure 1). Consequently, labeled bacteria could be used to further assess probiotic potential of the *L. plantarum* strains.

### *In vitro* Adhesion of *L. plantarum* M5MA1 and Its Derivatives to Caco-2 Cells

**FIGURE 3:**
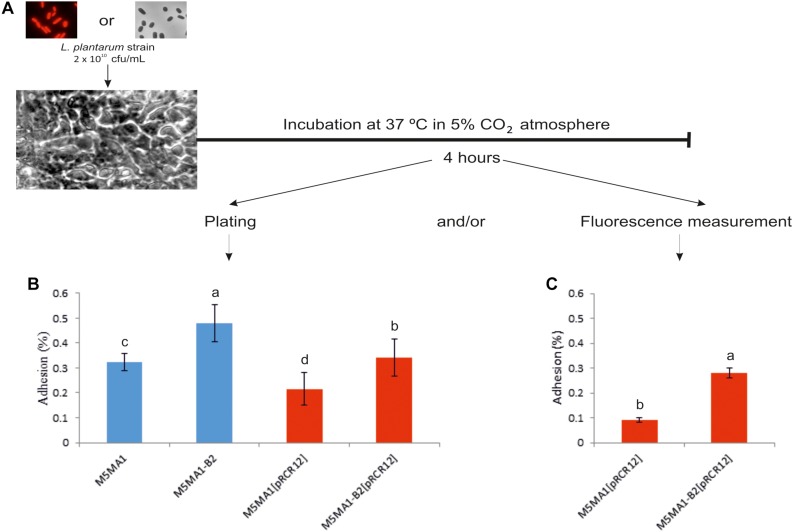
Adhesion of *L. plantarum* M5MA1 and derivative strains to Caco-2 cells. **(A)** Diagram of the experimental protocol. **(B)** BAL adhesion levels detected by plate counting. **(C)** BAL adhesion levels detected by mCherry fluorescence measured at 610 nm upon excitation at a wavelength of 587 nm. The standard curves depicted in Supplementary Figure S2 were used to correlate mCherry fluorescence and number of cfu. Statistical significances are represented by different letters that mean *P* ≤ 0.01.

The capacity to adhere to the intestinal epithelium is one of the properties sought in probiotic bacteria. Therefore, the ability of the *L. plantarum* M5MA1 and its three derivative strains to adhere Caco-2 human cells was investigated following the protocol depicted in Figure 3A. When levels of bound bacteria were determined by plate counting both riboflavin-overproducing M5MA1-B2 and M5MA1-B2[pRCR12] strains showed a significant 1.5-fold higher adhesion than those of their corresponding riboflavin-producing strains M5MA1 and M5MA1[pRCR12] (Figure 3B). Moreover, the presence of the pRCR12 plasmid in both the wild-type strain and the B2 mutant resulted in a decrease of 1.4-fold decrease of the adhesion levels (Figure 3B). Fluorescence staining of LAB (Bianchi et al., 2004) and fluorescent labeling of bifidobacteria (Castro-Bravo et al., 2017) have been used to quantify their capacity to adhere to enterocytes *in vitro*. Therefore, the mCherry fluorescence was used to quantify the adhesion of the *L. plantarum* strains carrying the pRCR12 plasmid. Standard curves of the two strains correlating cfu and fluorescence were used for quantification (Supplementary Figure S2). The levels of adhesion of both strains (Figure 3C) were slightly lower than that detected by direct platting (Figure 3C) and again a higher binding level (3.1 fold) of M5MA1-B2[pRCR12] versus M5MA1[pRCR12] was observed. Thus, the overall above results revealed that the fluorescence of *L. plantarum* strains labeled with pRCR12 could be used to quantify their adhesion to Caco-2 cells. Moreover, they reveal that *L. plantarum* M5MA1-B2[pRCR12] can be used instead of M5MA1-B2 for testing in GIT models.

### Performance of *L. plantarum* M5MA1-B2[pRCR12] in the BFBL Gut Model

**FIGURE 4:**
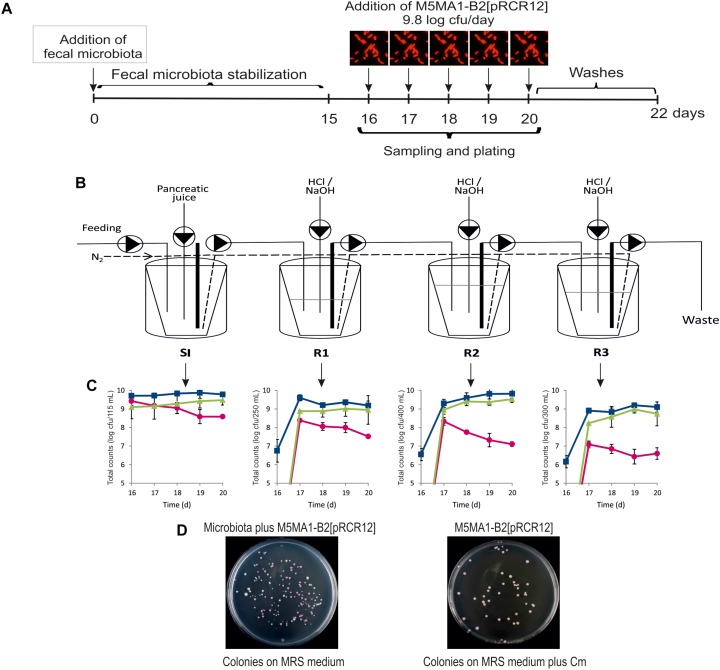
Performance of *L. plantarum* M5MA1-B2[pRCR12] in an *in vitro* model of human intestinal tract. **(A)** Diagram of the experimental protocol. **(B)** Schematic representation of the BFBL gut model (SI, small intestine; R1, proximal colon; R2, transverse colon; R3, distal colon). **(C)** Detection of LAB in the compartments of the BFBL model by qPCR (*Lactobacillus*, blue; *L. plantarum* strains carrying *ribG* gene, green) and plate counting (*L. plantarum* M5MA1-B2[pRCR12], fucsia). **(D)** Detection of M5MA1-B2[pRCR12] on plates by the pink color of its colonies in presence (left plate) or absence (right plate) of microbiota.

The above results showed that *L. plantarum* M5MA1-B2[pRCR12] could bind to Caco-2 human cells, but prior to the adhesion the bacteria have to reach the enterocytes location. Therefore, to test the capacity of the bacteria to reach and remain in the gut, the survival of this bacterium was evaluated after the passage through the simulated GIT conditions including the dynamic simulation of the human gut microbiota following the protocol depicted in Figure 4A and by using the BFBL gut model depicted in Figure 4B. Samples were taken during the test period and the microbial counts obtained for the SI and R1, R2, and R3 reactors are shown in Figure 4C. The fluorescent labeling of M5MA1-B2[pRCR12] permitted the straightforward determination of the strain viability by counting pink colonies detected alone when grown on MRS-agar medium supplemented with Cm, or clearly distinguishable among the rest of white colonies corresponding to members of the human microbiota, when plated on MRS-agar medium (Figure 4D). In average, after feeding 5 days the BFBL simulator with M5MA1-B2[pRCR12] at 9.8 log cfu/day (suspended in nutritional medium adjusted to pH 2, to resemble the acidic stress of the stomach), the SI released to the colon reactors about 8.96 log cfu/day during all the feeding period, indicating a satisfactory resistance of the strain to gastric and intestinal conditions. Pink colonies were observed in the plating analysis of the colon reactors content only during the feeding period with *L. plantarum* M5MA1-B2[pRCR12] (Figure 4C and data not shown). The pattern of detection of the *L. plantarum* strain by MRS-agar plating indicated a gradual reduction of viability during its colonic transit, with counts of 6.8 log cfu/day in the reactor simulating the distal colon. Moreover, the pink colonies disappeared in the three colon reactors after the 3-days washout period (results not shown), indicating a low colonization capability of *L. plantarum* M5MA1-B2[pRCR12] in the BFBL simulator of human microbiota. Feeding the BFBL model with *L. plantarum ribG* increased accordingly the *Lactobacillus* and *ribG* counts obtained by qPCR (Figure 4C), whereas total bacteria counts showed not change between the stabilization and test periods (results not shown). No *ribG* gene was detected during the stabilization period, indicating low presence of riboflavin producing *L. plantarum* in the donors’ fecal microbiota. However, some *L. plantarum* strains, such as the strain model WCFS1, contain an incomplete *rib* operon devoid of the entire *ribG* gene and it is unable to produce riboflavin (Kleerebezem et al., 2003). Therefore, the lack of detection of the *ribG* gene could be due to the presence of non-riboflavin producing *L. plantarum* strains. Nevertheless, this result confirms as previously proposed that the *ribG* presence represents a good marker for detection of riboflavin-producing bacteria (Burgess et al., 2006).

Besides monitorization of the riboflavin producing strain, products of fermentative metabolism in the colon reactors were also quantified: SCFA (acetic, propionic, butyric and formic acids) as well as lactic acid (fermentative metabolism) and ammonium (derived from proteolytic metabolism). The values detected during the stabilization and test periods are shown in the Supplementary Table S1. Lactate was determined in the three colon reactors only during all the experimentation with one volunteer fecal sample and only in the R1 vessel during the second one. In general, the feeding of the BFBL simulator with *L. plantarum* M5MA1-B2[pRCR12] resulted in no significant changes (*p* > 0.05) of neither SCFA nor ammonium contents. Therefore, this result indicates that the transient presence of the riboflavin producing strain did not affect the global metabolism of the human microbiota. In addition, the overall results showed a low colonization capability of *L. plantarum* M5MA1-B2[pRCR12], in spite of a good resistance to the acidic and bile salt conditions. Van den Abbeele et al. (2012) demonstrated that incorporating a mucosal environment in the SHIME dynamic gut model resulted in a more representative colonization by lactobacilli, particularly for strong mucin-adherent *Lactobacillus* strains. Consequently, the low colonization of *L. plantarum* M5MA1-B2[pRCR12] in the BFBL gut model could be due to the absence of enterocytes and mucin, that will be present in an *in vivo* model.

### Survival of *L. plantarum* M5MA1-B2[pRCR12] in the Mouse GIT

**FIGURE 5:**
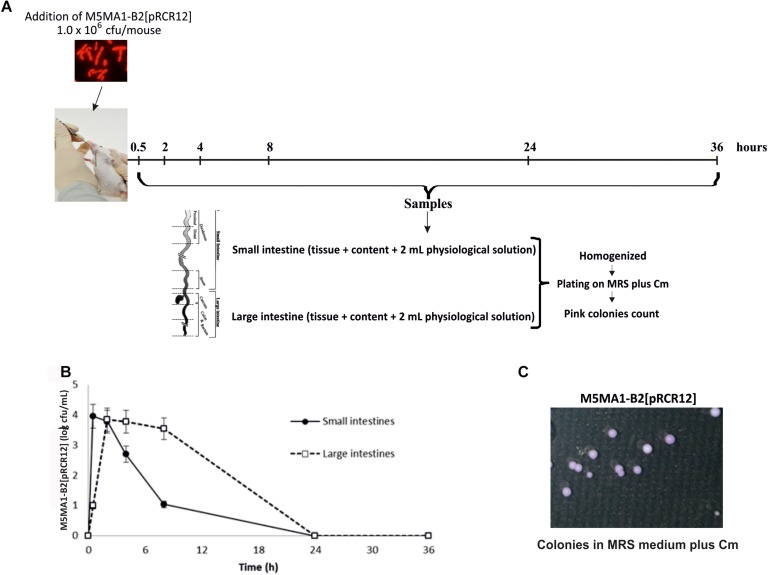
Performance of *L. plantarum* M5MA1-B2[pRCR12] in mouse digestive tract. **(A)** Diagram of the experimental protocol. **(B)** M5MA1-B2[pRCR12] recovered from mice intestines by plating. **(C)** M5MA1-B2[pRCR12] red colonies detected after plating of a sample from small intestine taken 4 h after LAB administration in MRS supplemented with Cm.

The above results and the fact that *L. plantarum* 423 strain chromosomally labeled with the mCherry coding gene was successfully investigated in a GIT mouse model (van Zyl et al., 2015) prompted us to test *L. plantarum* M5MA1-B2[pRCR12] in an *in vivo* murine model following the protocol depicted in Figure 5A. *L. plantarum* M5MA1-B2[pRCR12] was detected in the small intestines healthy BALB/c conventional mice until 24 h post-administration (Figure 5B), being the highest value (4.0 ± 0.3 cfu/mL) detected at 30 min after bacterial feeding. In the large intestines smaller numbers of the bacterium were found at 30 min (1.0 ± 0.2 cfu/mL) and peaked between 2 and 8 h post-incubation (3.5–3.9 cfu/mL). Analysis of mouse samples by plating revealed the presence of the bacterium by the pink color of its colonies (Figure 5C). No pink colonies were found in the control mice that were administered with saline solution. From these results it can be inferred that although there was a loss of bacterial viability during the passage from the stomach to the intestines (i.g. administration of log 6.0), large numbers were recovered in the small intestines 30 min after administration and in the large intestines starting at 2 h post-administration. These results confirm the *in vitro* studies that showed that the strain was able to resist the conditions of the gastrointestinal tract. Since the strain was not recovered after 24 h post-administration, it can be concluded that although the strain can survive the passage through the gastrointestinal tract, it cannot colonize in mice. These results suggest that continuous administration of the strain (on a daily basis) would be required to exert a biological activity (produce riboflavin) at the site of interest (small intestines where the vitamin would be absorbed). Probiotic colonization is usually low, but important is the activity as transient metabolism or host interaction. In any case, the low colonization of *L. plantarum* M5MA1-B2[pRCR12] in the *in vivo* model was in accordance with the results obtained in the *in vitro* BFBL gut model.

## Conclusion

We have optimized a direct fluorescence spectroscopic quantitative detection method of vitamin B2 that could be useful for a fast selection of riboflavin overproducing LAB. Labeling with the pRCR12 plasmid expressing the red fluorescent mCherry protein has generated an attractive tool to evaluate the survival of candidate probiotic strains under digestive tract conditions in the presence of microbiota in the dynamic multistage BFBL gut model, and to quantify the adhesion level to Caco-2 human cells. Moreover, this construction has been successfully employed to track probiotic *L. plantarum* strains within the gut by using a murine model and bacterial platting. Therefore, the proposed approaches could be implemented, by using both *in vitro* and *in vivo* models as a workflow to select *L. plantarum* strains with application in the field of functional foods.

## Data Availability

Publicly available datasets were analyzed in this study. This data can be found at: https://www.ncbi.nlm.nih.gov/nuccore/NZ_CAADEV000000000.1.

## Ethics Statement

Conventional adult BALB/c mice (male, 5 weeks old, weighing 25 ± 3 g) were obtained from the inbred animal facilities at the Centro de Referencia para Lactobacilos (CERELA-CONICET, San Miguel de Tucumán, Argentina). The animal protocol was pre-approved by the Animal Protection Committee of the CERELA (protocol no. CRL-BIOT-LT-20142/A), and all experiments complied with the current laws of Argentina for the use of experimental animals.

## Author Contributions

MM and SH-R performed all the *in vitro* analysis of the LAB. AY provided the background for handling the riboflavin-producing LAB. TR, MM-C, and CP contributed to the analysis of performance of LAB under digestive tract conditions in interaction with the human microbiome. JL performed the LAB analysis in the animal model. PR and GS were responsible for processing and the HPLC analysis of the riboflavin present in supernatants of bacterial and Caco-2 cultures, and the design of strategies to develop and analyze the bacterial recombinant strains and corrected the manuscript. GS, PL, and RA participated in the study conception, data interpretation, and generated the final version of the manuscript. All authors listed have read and approved the final version of the manuscript.

## Conflict of Interest Statement

The authors declare that the research was conducted in the absence of any commercial or financial relationships that could be construed as a potential conflict of interest.

## Footnotes

1 http://www.ebi.ac.uk/Tools/msa/clustalo
