# Supplementary material for: Real-Time Detection of Riboflavin Production by Lactobacillus plantarum Strains and Tracking of Their Gastrointestinal Survival and Functionality in vitro and in vivo Using mCherry Labeling
Source: Front Microbiol. 2019 Jul 31;10:1748. doi: 10.3389/fmicb.2019.01748 (PMC6684964; doi:10.3389/fmicb.2019.01748)
Supplement: Supplementary file 1 [file Data_Sheet_1.docx]

**Real-time detection of riboflavin production by *Lactobacillus plantarum* strains and tracking of their gastrointestinal survival and functionality *in vitro* and *in vivo* using mCherry labeling**

*Mari Luz Mohedano^1^, Sara Hernandez*-*Recio^1^, Alba Yepez^2^, Teresa Requena^3^, M. Carmen Martínez-Cuesta^3^, Carmen Peláez^3^, Pilar Cano^3^, Pasquale Russo^4^****, Jean Guy LeBlanc^5^,*** *Giuseppe Spano^4^****, Rosa Aznar^2,6*^, Paloma López^1^***

^1^Department of Microorganisms and Plant Biotechnology, Biological Research Center (CIB-CSIC), Madrid, Spain.

^2^Department of Microbiology and Ecology, University of Valencia, Burjassot, Spain.

**^3^**Institute of Food Science Research (CIAL-CSIC), Department [of Biotechnology and Food Microbiology](https://www.cial.uam-csic.es/en/research-and-innovation/departments/department-of-biotechnology-and-microbiology/)**, Madrid, Spain.**

**^4^**Department of the Science of Agriculture, Food and Environment, University of Foggia, Foggia, Italy.

**^5^Reference Centre for lactobacilli (CERELA-CONICET), Tucuman, Argentina.**

**^6^**Department of Preservation and Food Safety Technologies, Institute of Agrochemistry and Food Technology (IATA-CSIC), Paterna, Spain.

**Supplementary Figure S1**. **Riboflavin calibration curve.** Correlation of riboflavin concentration and fluorescence. Serial dilutions of a riboflavin solution in CDM medium lacking riboflavin at 10 mg/mL were used to determine its fluorescence emission at a wavelength of 520 nm after excitation at a wavelength of 440 nm.


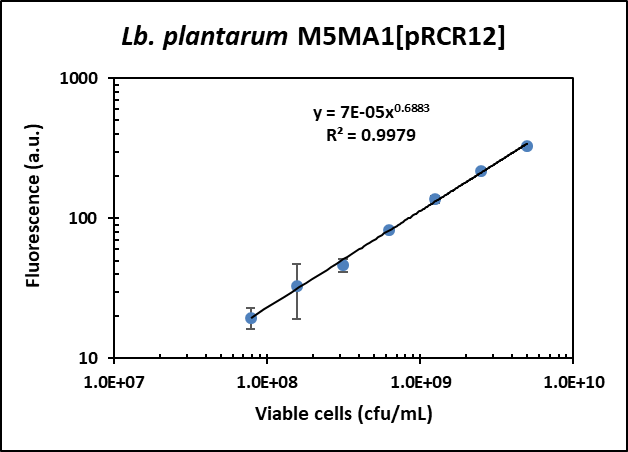

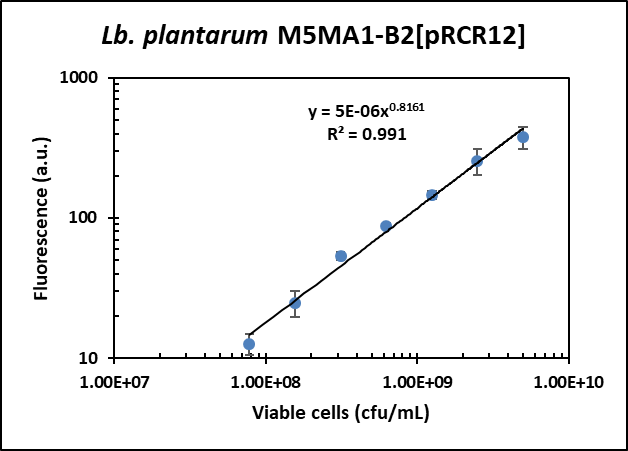


**Supplementary Figure S2. Calibration curve of viable cells.** After removal of culture supernatants, serial dilutions of bacterial cultures resuspended in PBS at 1 x 10^10^ cfu/mL determined by plating were diluted and the emission of the mCherry fluorescence expressed in the bacteria was measured at a wavelength of 610 nm after excitation at a wavelength of 587 nm.

**Wild-type CGATTTCTTCGGGGCAGGGTGCAATTCCCGACCGACGGTAACAACGTAAGTTGAAGTCCGTGACCCGCGTGAGCGGTGGACCCAGTGCAAGTCTGGGACCGACAGTATAGTCTGGATGGGAGAAGAAAATT**

**M5MA1-B2 CGATTTCTTCGGGGCAGGATGCAATTCCCGACCGACGGTAACAACGTAAGTTGAAGTCCGTGACCCGCGTGAGCGGTGGACCCAGTGCAAGTCTGGGACCGACAGTATAGTCTGGATGGGAGAAGAAAATT**

**M9MM1-B2 CGATTTCTTCGGGGCAGGATGCAATTCCCGACCGACGGTAACAACGTAAGTTGAAGTCCGTGACCCGCGTGAGCGGTGGACCCAGTGCAAGTCTGGGACCGACAGTATAGTCTGGATGGGAGAAGAAAATT**

**M9MG6-B2 CGATTTCTTCGGGGCAGGCTGCAATTCCCGACCGACGGTAACAACGTAAGTTGAAGTCCGTGACCCGCGTGAGCGGTGGACCCAGTGCAAGTCTGGGACCGACAGTATAGTCTGGATGGGAGAAGAAAATT**

**M9Y2-B2 CGATTTCTTCGGGGCAGGGTGCAATTCCCGACCGACGGTAACAACGTAAGTTGAAGTCCGTGACCCGCGTGAGCGGTGGACCCAGTGCAAGTCTGGGACCGACAGTATAGTCTAGATGGGAGAAGAAAATT**

**M9MM4-B2 CGATTTCTTCGGGGCAGGGTGCAATTCCCGACCGACGGTAACAACGTAAGTTGAAGTCCGTGACCCGCGTGAGCGGTGGACCCAGTGCAAGTCTGGGACCGACAGTATAGTCTGGATGGGAGAAAAAAATT**

******************** ********************************************************************************************** ********** ********

**Supplementary Figure S3**. **DNA sequence of the RFN regions of the *L. plantarum* strains**. The identical sequences of the wild-type strains (wild-type) as well as of those of their derivatives are depicted. Nucleotides with yellow background indicate the mutations detected in the riboflavin-overproducing derivatives.

**
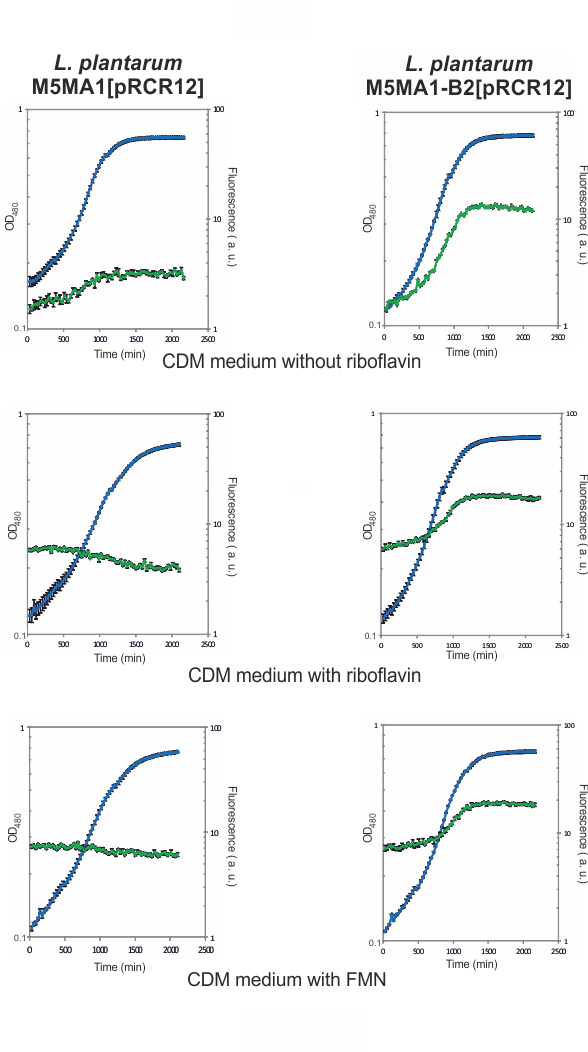
**

**Supplementary Figure S4.** **Detection of riboflavin production by *L. plantarum* M5MA1[pRCR12] and M5MA1-B2[pRCR12] during growth.** Bacteria were grown in CDM medium without riboflavin, or supplemented with either riboflavin or FMN both at a concentration of 2 µg/mL. The growth of cultures (blue) was monitored by measurement of OD_480_. Fluorescence emission of riboflavin or FMN (green) was recorded at 520 nm after excitation at a wavelength of 440 nm.

**Supplementary Table S1. Detection of SCFA and ammonium concentration in the vessels of the BFBL**

| Compound | Sample | R1 | R2 | R3 |
| --- | --- | --- | --- | --- |
| Acetate | Stab | 37.56±7.95 | 49.72±8.79 | 55.41±12.57 |
|  | Test | 39.95±7.07 | 48.876.35 | 54.01±13.22 |
| Propionate | Stab | 10.95±3.70 | 17.65±4.16 | 16.27±4.72 |
|  | Test | 10.08±2.22 | 17.42±v | 16.99±4.75 |
| Butyrate | Stab | 2.18±1.32 | 4.13±0.86 | 2.98±1.39 |
|  | Test | 2.63±1.25 | 4.16±0.85 | 3.00±1.11 |
| Lactate* | Stab | 1.91±0.17 | 0.920.15 | 0.73±0.12 |
|  | Test | 1.51±0.24 | 0.83±0.05 | 0.77±0.09 |
| Formate | Stab | 0.62±0.04 | 0.47±0.03 | 0.43±0.02 |
|  | Test | 0.76±0.17 | 0.50±0.04 | 0.45±0.02 |
| Ammonium | Stab | 6.70±0.32 | 11.37±0.98 | 13.64±0.20 |
|  | Test | 6.81±0.82 | 11.08±1.11 | 13.41±1.78 |

*One inoculum only in R1, with stabilization value 0.79±0.03 and 0.67±0.15 during the test period. No changes in SCFA and ammonium values (Student's *t*-test) between estabilization (Stab) and test periods.
